# Correction to ‘The chromatin accessibility dynamics during cell fate specifications in zebrafish early embryogenesis’

**DOI:** 10.1093/nar/gkae1286

**Published:** 2024-12-19

This is a correction to: Qiushi Xu, Yunlong Zhang, Wei Xu, Dong Liu, Wenfei Jin, Xi Chen, Ni Hong, The chromatin accessibility dynamics during cell fate specifications in zebrafish early embryogenesis, *Nucleic Acids Research*, Volume 52, Issue 6, 12 April 2024, Pages 3106–3120, https://doi.org/10.1093/nar/gkae095

In the version originally published, the grant number for funding from the Shenzen Science and Technology Program was incorrectly given as 202208150943300001. This has been corrected to read 20220815094330001.

