# Supplementary material for: The chromatin accessibility dynamics during cell fate specifications in zebrafish early embryogenesis
Source: Nucleic Acids Res. 2024 Feb 14;52(6):3106–20. doi: 10.1093/nar/gkae095 (PMC11014328; doi:10.1093/nar/gkae095)
Supplement: gkae095_Supplemental_Files [file gkae095_supplemental_files.zip › supplementary_figures.pdf]

A

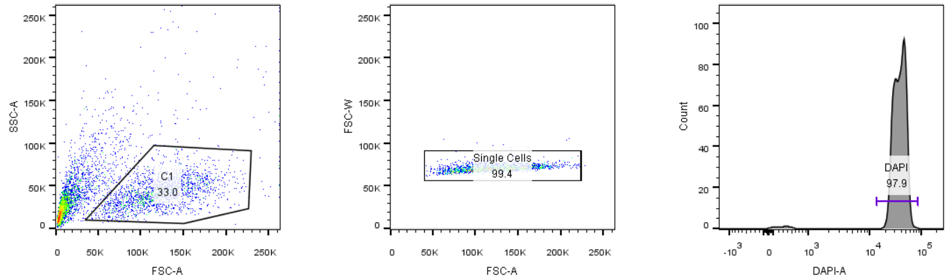

B

stage	nCount_peaks	nFeature_peaks	dup_level	library_size	mt_content	sequencing_depth	mapping_rate	frac_open	frip	uniq_nuc_frags	nucleosome_signal	TSS.enrichment
**1k**	20148.5	16441.5	0.729869	33246.0	0.856454	194282.0	75.730	4.32055	50.29120	31058.0	1.259904	2.243089
**high**	40575.0	32365.0	0.575767	71273.5	0.277550	255999.5	77.290	8.08420	55.63945	61610.5	0.875769	2.675763
**oblong-sphere**	21125.5	15767.5	0.690079	29273.0	0.796164	154448.5	76.960	4.92200	56.62405	26767.5	1.735721	3.119811
**sphere-dome**	27246.0	22121.5	0.693064	41071.5	0.720137	204490.0	80.795	7.77955	54.45175	36909.0	1.792453	3.154337
**dome**	29439.0	22766.0	0.706239	44789.0	0.665239	230776.5	80.800	8.39785	56.87360	38540.0	1.756306	3.231722

C

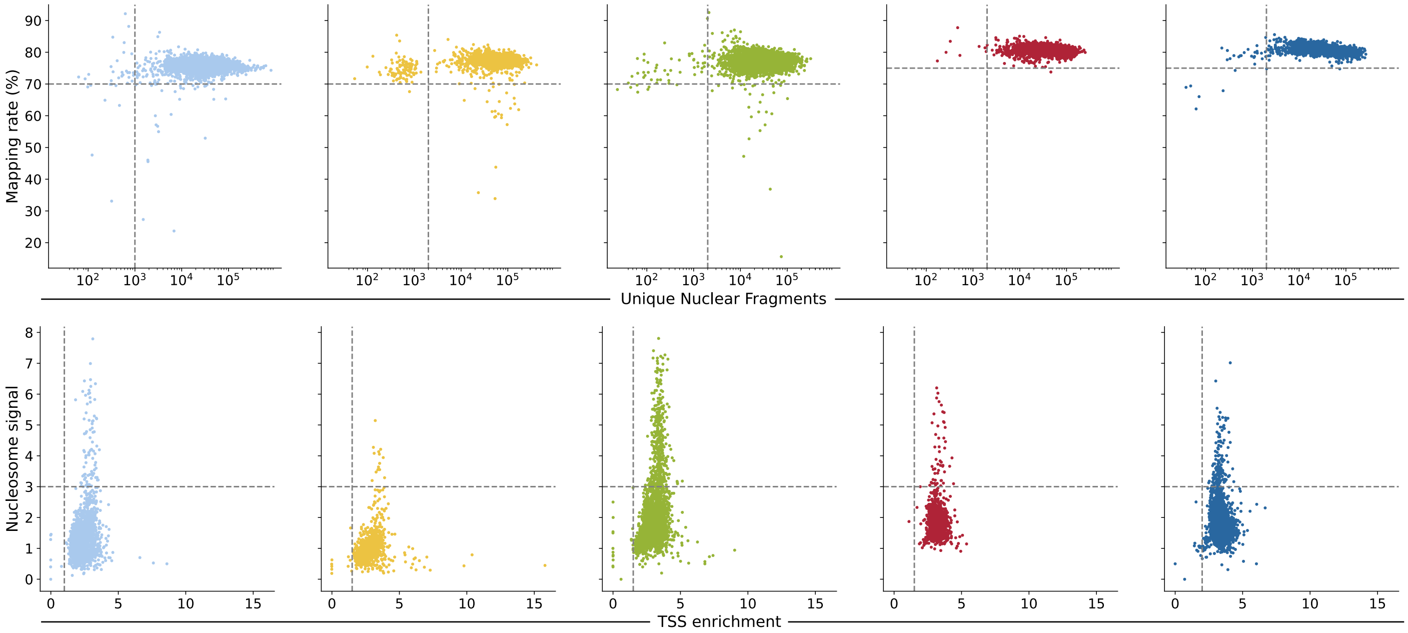

D

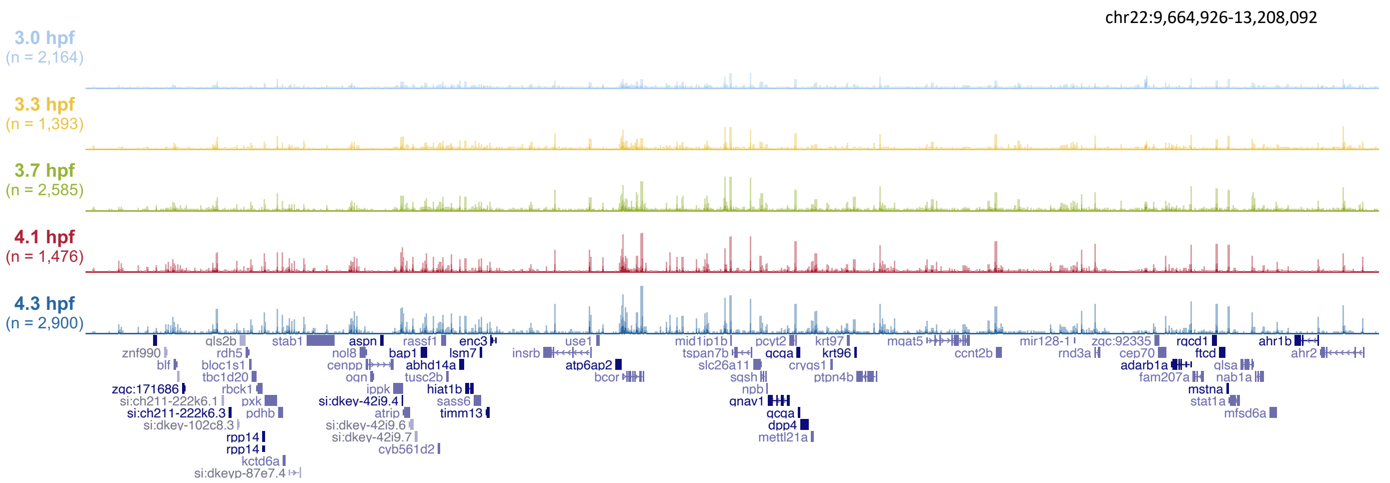

Supplementary Figure 1 | Quality control (QC) of the whole single-nucleus ATAC-seq experiment. (**A**) Examples of FACS for a typical experiment. FSC-A and SSC-A were used to remove cell debris. FSC-A and FSC-W were used to select only single nucleus. DAPI+ events were used to select only successfully permeabilised cells. (**B**) Median information of each QC metric in different stages. (**C**) QC cutoff used to remove failed wells in the experiment. (**D**) UCSC genome browser tracks from the indicated genomic locus showing an overview of the data quality.

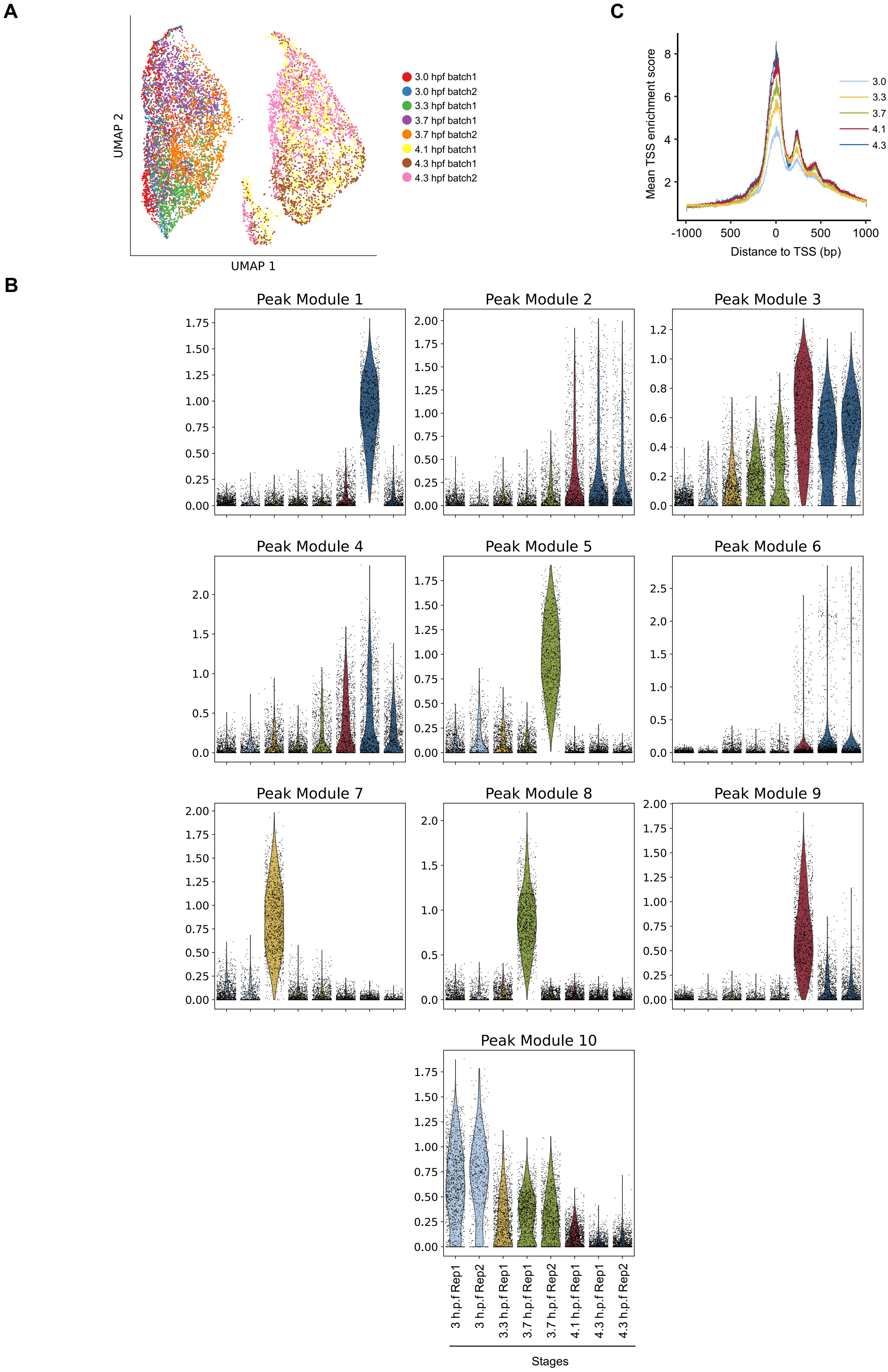

Supplementary Figure 2 | The overview of the entire single nucleus ATAC-seq data set. (**A**) BBKNN integrated UMAP of all nucleus, coloured by experimental batches. (**B**) The module weights of each peak module grouped by experimental batches. (**C**) The global TSS enrichment scores of each time point.

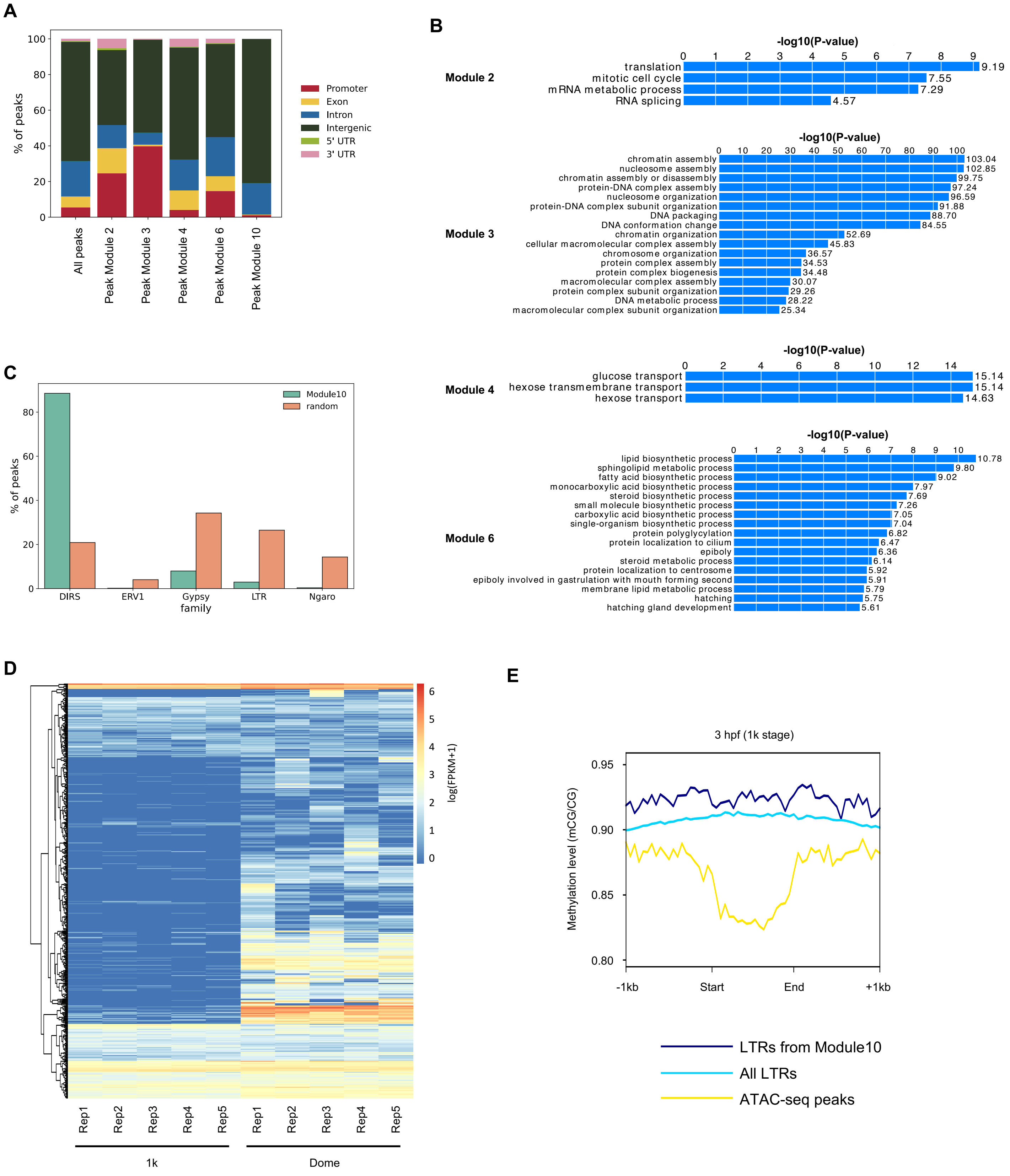

Supplementary Figure 3 | Different genomic features and functions of each peak module. (**A**) The genomic distribution relative to annotated genes of each peak module. (**B**) GREAT analysis of enriched biological processes in each peak module. (**C**) The distribution of the number of peaks in peak module 10 that overlapped the indicated LTR family. Random was the background, which contained 2,000 randomly selected peaks from all ATAC-seq peaks. (**D**) The expressions of 519 LTRs in module 10 at 1k and dome stages. Data were taken from White *et al.* 2017 (54). (**E**) The DNA methylation levels (mCG/CG) at 3 hpf (1k stage) around indicated regions. 1 kb upstream and downstream were also plotted. Data were taken from Ross *et al.* 2023 (56).

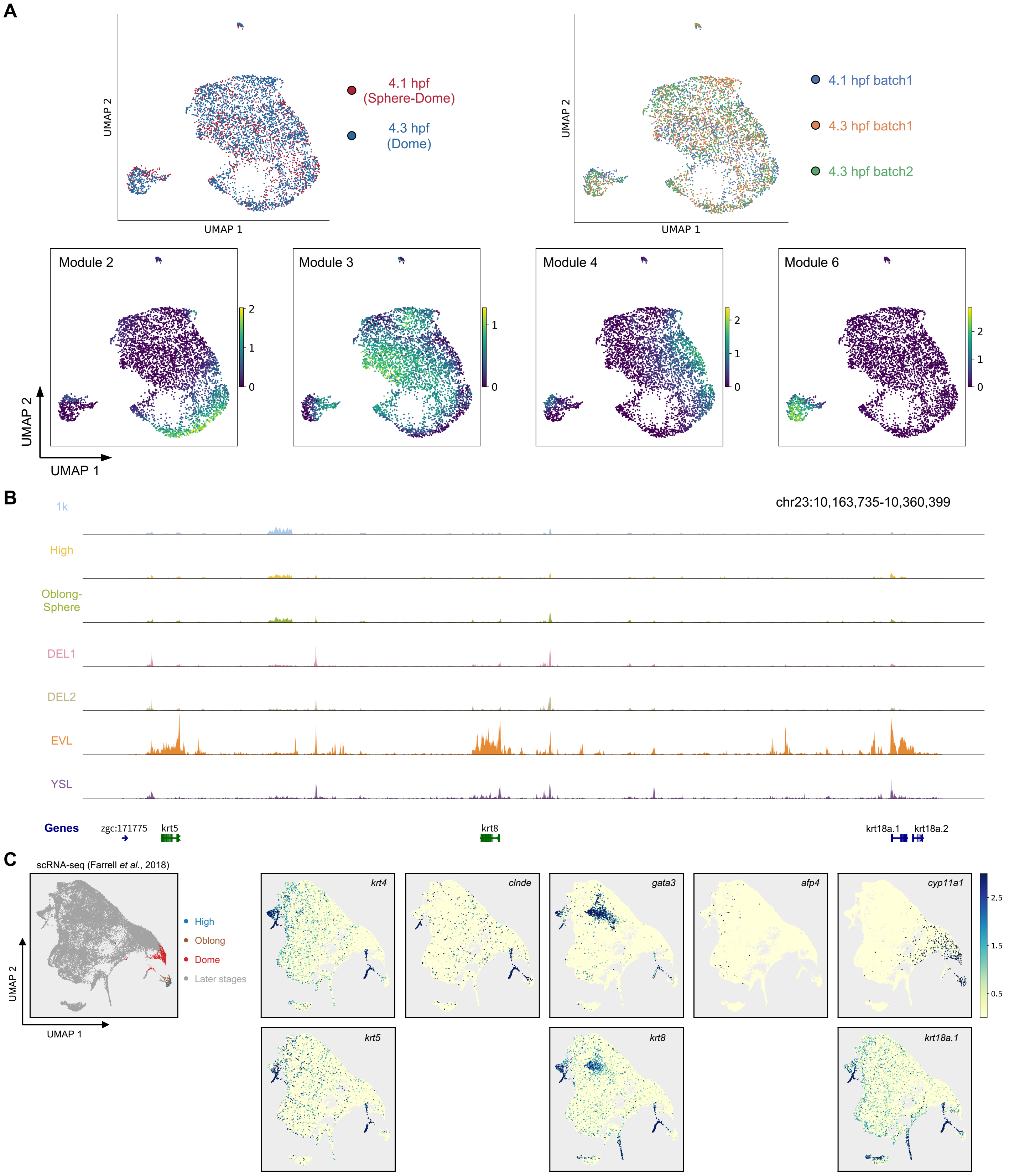

Supplementary Figure 4 | Analysis of nucleus from 4.1 hpf (sphere-dome) and 4.3 hpf (dome) stages. (**A**) The same UMAP plot as in **Figure 4A**, coloured by time point (top left), experimental batches (top right) and module weights (bottom). (**B**) Browser tracks showing specific peaks around three EVL marker genes. (**C**) The expression levels of indicated genes from a previously published scRNA-seq atlas.

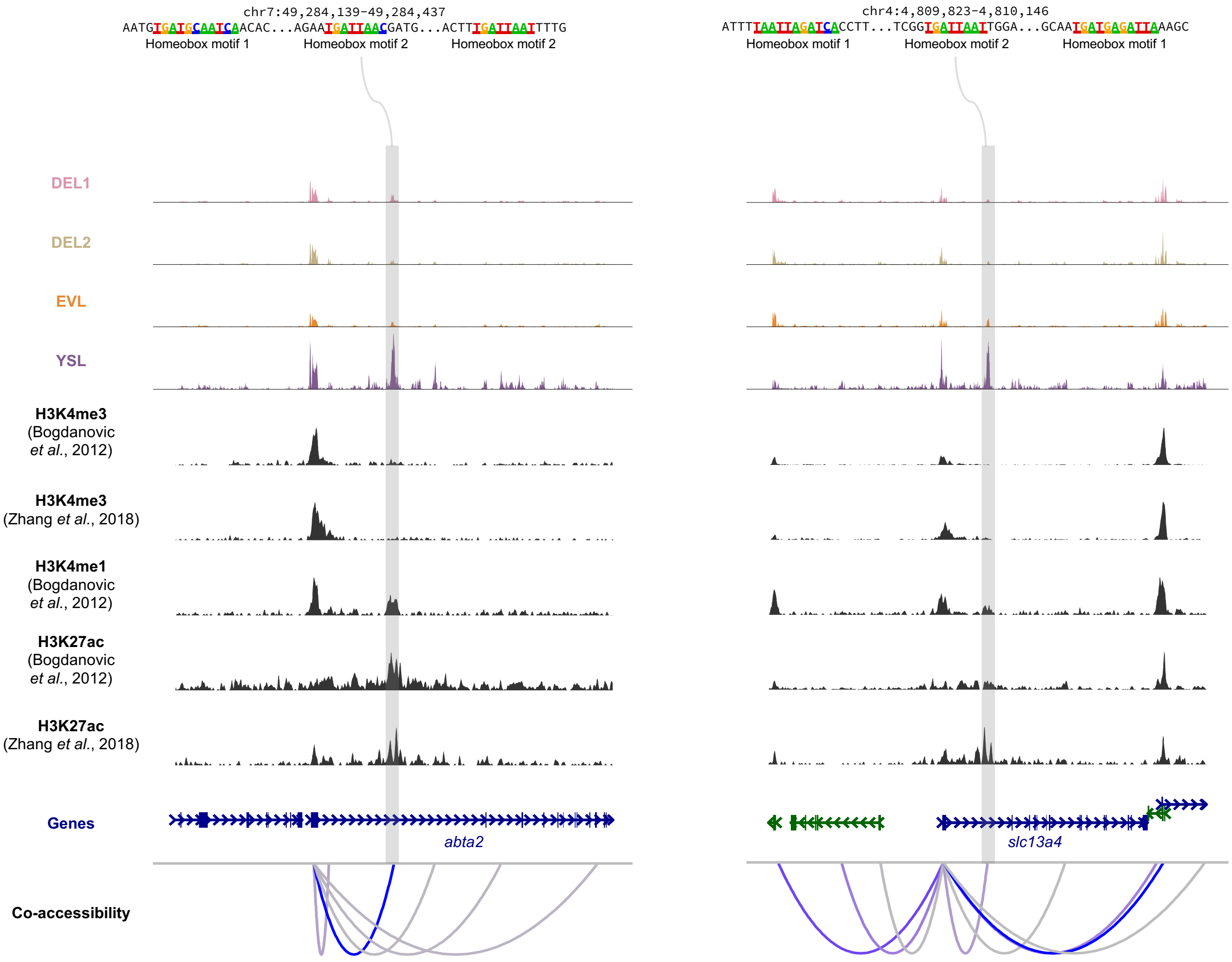

Supplementary Figure 5 | Examples of peak co-accessibility around two YSL marker genes. Cell-type specific open chromatin peaks with motifs from **Figure 5C** were highlighted. ChIP-seq data of H3K4me3, H3K4me3 and H3K27ac from two previous studies were also shown.

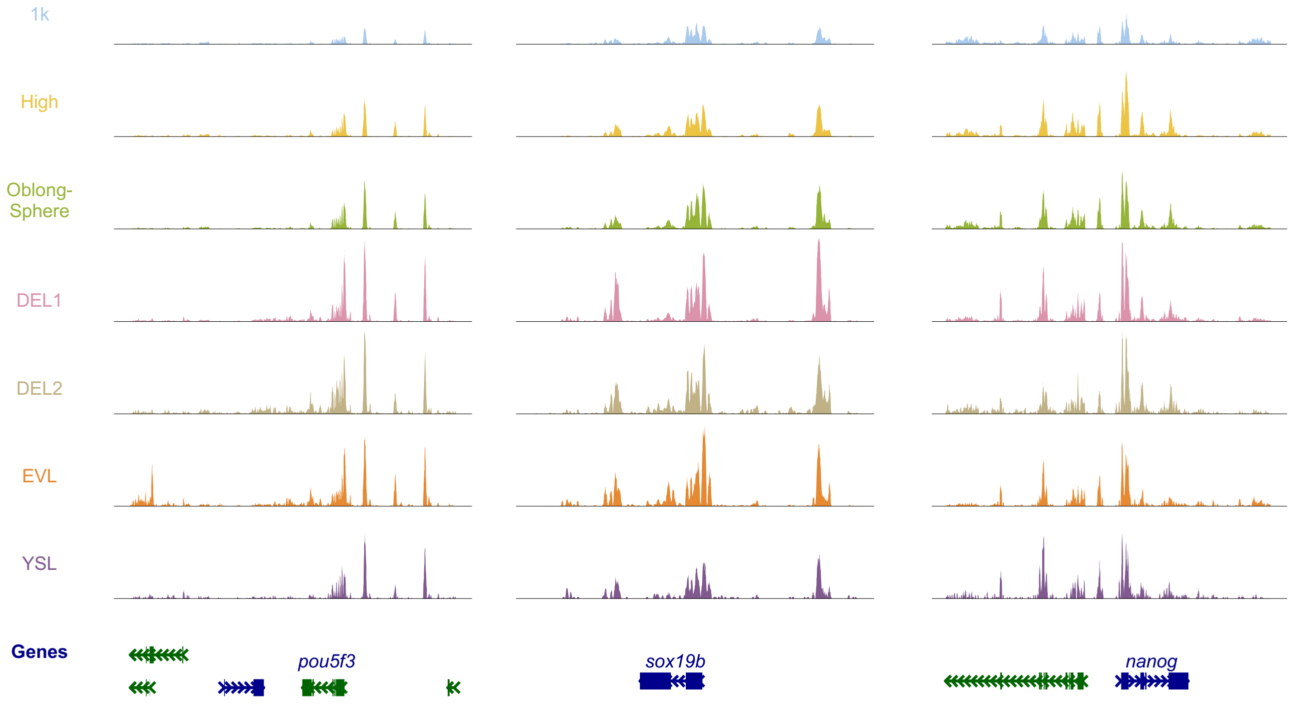

Supplementary Figure 6 | Browser tracks showing the aggregated single-nucleus ATAC-seq signal in each cell cluster around the three pluripotent genes.
