# Supplementary material for: The chromatin accessibility dynamics during cell fate specifications in zebrafish early embryogenesis
Source: Nucleic Acids Res. 2024 Feb 14;52(6):3106–20. doi: 10.1093/nar/gkae095 (PMC11014328; doi:10.1093/nar/gkae095)
Supplement: gkae095_Supplemental_Files [file gkae095_supplemental_files.zip › Supplementary_Table_Legends.docx]

Supplementary Table Legends

Supplementary Table 1 | Metadata and quality metrics for all single cells in the study, including both successful and failed cells.

Supplementary Table 2 | Module weights in each successful cells for all 25 peak modules. This is first matrix returned by the non-negative matrix factorisation analysis.

Supplementary Table 3 | Marker peaks in each of the cell cluster from the combined 4.1 and 4.3 hpf analysis. This the result from the differential accessibility test by Signac. The results for different cell clusters are organised into different tabs.

Supplementary Table 4 | The raw data for the heatmap of Figure 5F, containing the Z score of each homeodomain transcription factor in the indicated cell cluster.
